# The burden of stroke in China: Results from a nationwide population-based epidemiological survey

**DOI:** 10.1371/journal.pone.0208398

**Published:** 2018-12-06

**Authors:** Yilu Gao, Bin Jiang, Haixin Sun, Xiaojuan Ru, Dongling Sun, Linhong Wang, Limin Wang, Yong Jiang, Valery L. Feigin, Yilong Wang, Wenzhi Wang

**Affiliations:** 1 Department of Neuroepidemiology, Beijing Neurosurgical Institute, Capital Medical University, Beijing, China; 2 Beijing Municipal Key Laboratory of Clinical Epidemiology, Beijing, China; 3 National Center for Chronic and Non-communicable Disease Control and Prevention, Chinese Center for Disease Control and Prevention, Beijing, China; 4 Beijing Tiantan Hospital, Capital Medical University, Beijing Institute for Brain Disorders, Beijing, China; 5 National Institute for Stroke and Applied Neurosciences, School of Public Health and Psychosocial Studies, Faculty of Health and Environmental Sciences, Auckland University of Technology, Auckland, New Zealand; Faculty of Science, Ain Shams University (ASU), EGYPT

## Abstract

Stroke is a serious threat to human health that often leads to severe complications, and currently ranks first as leading cause of death in China. However, reliable data on stroke burden in China in the 21^st^ century are lacking. We used the data from NESS-China (National Epidemiological Survey of Stroke in China) for assessing the adverse health effects of stroke in Chinese population. We carried out inter-regional comparative study in order to obtain regular burden related characteristics of stroke in China, as measured by YLLs (years of life lost due to premature mortality), YLDs (years lived with disability) and DALYs (disability adjusted life years). Amongst the nationwide population of 596,536 individuals of all ages in 2013, the YLLs for stroke was 1748, the YLDs was 262, and the DALYs was 2010(per 100,000). The gender subtype analysis of DALYs was 2171(male) and 1848(female). The YLLs, YLDs and DALYs in rural areas were higher compared to urban areas. Among the 18 age groups, the highest YLLs was observed in ≥ 80 years old group. The impact of stroke on Chinese population is more severe compared to the global average levels. Stroke results as the main cause of YLLs in China, while there is no significant difference for the YLDs. Nevertheless, DALYs caused by stroke rank 3^th^ in global epidemiologic study territories, 1^st^ in China.

## Introduction

Currently, stroke is the leading cause of death in China[1, 2], and its adverse consequences to the physical health are becoming ever more serious[3, 4]. Conducting epidemiological studies and clarifying the burdens associated with this disease are crucial for establishing prevention and control strategies, and for evidence-based allocation of public health resources[5–7]. However, reliable data on stroke burden in China in the 21^st^ century are lacking.

During “The Twelfth Five-year Plan” (2011–2015), National Ministry of Science and Technology and National Health Planning Commission instigated the program of “National Epidemiological Survey of Stroke in China (NESS-China)”, and carried out an epidemiological survey on cerebrovascular disease at 155 different survey sites in 31 provinces. The scope of this initiative was to acquire the basic information regarding the current level of burden from and trends of cerebrovascular diseases in China, the basic risk factors, as well as the prevention and treatment of stroke in Chinese people. This epidemiological survey involved the largest sample scale in the field of cerebrovascular diseases since the foundation of People’s Republic of China [8]. The present research utilized the data from this survey especially focusing on the burden of stroke in China. The current health impact caused by stroke in relation to stroke induced disability and premature deaths were considered. Furthermore, socio-economic factors, the inter-regional differences in development status, different levels of public medical facilities and different geographical and climatic conditions were also considered in order to acquire a full comprehension of the factors influencing the burden of stroke in China.

## Methods

### Sampling method

NESS-China includes data from 2010 census[9] that represent current characteristics of Chinese rural and urban residents. These results were obtained using multilevel cluster random sampling method at 157 survey sites in 31 provinces (autonomous regions, municipalities). The National Disease Surveillance systems[1, 10] of the National Center for Chronic and Non-communicable Disease Control and Prevention of Chinese Center for Disease Control and Prevention, sampled at least 4,500 people at each survey site, and included 3,800 surveys (response rate ≥80%). The total sample size of the study population was 600,000[8].

### Ethical approval

The study was approved by the ethical review committees of Beijing Tiantan Hospital and all other participating institutes. Written informed consent was obtained from all study participants by interviewers before data collection. We can confirm that all methods were performed in accordance with the relevant guidelines and regulations.

### Diagnostic criteria

The stroke diagnostic criteria proposed by “Atherosclerosis Risk In Communities Study (ARIC)” were applied, such as “abruptly and rapidly evolutional focal/whole cranial nerve function defection, with the symptoms lasting more than 24 hours (unless surgical intervention is conducted or death is caused), without obvious reasons other than vascular factors, excluding abnormal nerve function caused by trauma, metabolic disturbance, poisoning, tumour or infection of central nervous system”[11].

The stroke mortality was determined by reviewing medical history (verbal autopsy), relevant medical records and neuroimaging examination materials when available.

Modified Rankin Scale (mRS)[12] was used to determine the sequela disability status in definite stroke cases[13].

### Procedures

The verification and inclusion of stroke sufferers and stroke mortalities was done via two–staged survey.[8] The first stage of the survey was done by trained investigators from the Centre for Disease Control who adopted face-to-face investigation approach at survey sites, regarding the household as the unit. They elucidated the relevant information for "cerebrovascular disease symptom screening and simple check-up" in the "Preliminary Screening Table of Epidemiology Survey of Cerebrovascular Diseases" to identify patients with suspected stroke and a history of stroke. They also obtained "family members death information" in the "Preliminary Screening Table" and death related information from the Centre for Disease Control during time period from September 1, 2012 to August 31, 2013. These data were submitted to the study neurologists for reviewing. At least 3,800 residents of all age groups at each study site completed the primary screening survey. The second stage of the survey was carried out primarily by professional neurologists who conducted face-to-face interviews and clinical/neurological examination of the study participants suspected to have a stroke, including reviewing of related medical records and neuroimaging data (when available) to confirm or refute the diagnosis of stroke. The relevant content was recorded in the "Registration Form of Definite Cases of Cerebrovascular Diseases" and included information on the onset time of stroke, sequelae disability status, duration of disability, and other relevant information. With reference to deceased, "Review Registration Form of Death Cases" was completed to determine the cause of deaths.

### Quality control

A close coordination and cooperation between National Research Group, the Provincial and Municipal/Survey Spot Center for Disease Control and Prevention, Provincial and Municipal/Survey spots for neurologists and other multiple centers and multiple sectors were established and maintained during the study. As a result, this survey established a nationwide three-level quality control mechanism, based on national, provincial, municipal, and survey sites, which implemented strict quality control for each study site and survey components.

### Statistical analysis

The Global Burden of Diseases, Injuries and Risk Factors (GBD) 2010[14] correlation methodology, and SPSS 17.0 Statistical Analysis Software were applied for data processing. All of the indices adjusted to the same standard world population composition as same as the GBD 2010 and had been estimated with 95% uncertainty interval (95%UI). The width of 95%UI provides a mechanism of communicating to users the limitations of estimates for the burden caused by different diseases, injuries and risk factors. Uncertainty around cause-specific YLLs, YLDs and DALYs were calculated incorporating uncertainty in levels of all-cause mortality, cause-specific mortality, prevalence, and disability weights.

YLLs were defined as total number of deaths (∑) due to stroke in **x**-age group multiplied by standard life expectancy in x-age group[15, 16]. In this study, all age groups were divided into 18 age groups: 0–364 days, 1–4 years old, 5–9 years old, 10–14 years old, 15–19 years old, 20–24 years old, 25–29 years old, 30–34 years old, 35–39 years old, 40–44 years old, 45–49 years old, 50–54 years old, 55–59 years old, 60–64 years old, 65–69 years old, 70–74 years old, 75–79 years old, and over 80 years old[17]. "∑" represented the cumulative sum of 18 age groups. The death of stroke in this study was limited to time period between September 1, 2012 to August 31, 2013.

YLDs were defined as total (∑) number of suffered from stroke sequela disability status in x-age group × corresponding weight of disability × duration of disability(year)[12, 15] In this study we adopted 220 types of classification standards resulted from 289 disabling diseases-1160 sequelae analyzed by GBD 2010. Disability sequel status of stroke were divided into four categories: mild disability, moderate disability, severe disability and critical disability, respectively corresponding to the modified Rankin Scale (mRS) description[13]. This study was limited to the stroke patients born before August 31, 2013. The duration of disability was defined from the onset of stroke leading to disability to August 31, 2013 (confirmed cases of survival) and the duration from the onset time leading to disability to death time-point (before 31 August 2013) (including those who died from other diseases but suffered from stroke previously). “∑” represented the cumulative sum of four types of sequela related disability.

DALYs (disability adjusted life years) were defined as YLLs+ YLDs[12, 15, 16].

## Results

NESS-China has completed preliminary screening of 596,536 cases among permanent residents in all age groups at 155 survey sites (from 157 Disease Surveillance Points) in 31 provinces[8, 18], among whom 7,030 cases were diagnosed with disability due to stroke before August 31, 2013, and 758 patients died as a result of stroke during September1, 2012 and August 31, 2013 (Table 1).

**Table 1:** The provincial detail of the population of disabled and death due to stroke and the absolute value of YLLs, YLDs and DALYs of stroke in all age groups obtained by NESS-China at 155 survey sites in 31 provinces nationwide*.

Provinces	Disabled (n)	Death (n)	YLLs (95%UI)	YLDs (95%UI)	DALYs (95%UI)	DALYs per 100,000 (95%UI)	2013 GDP per capita (U.S. Dollar)[26]
Tianjin	205	17	235(221–258)	75(61–98)	310(296–333)	3846(3674–3929)	15761
Xizang	30	13	259(252–268)	14(7–23)	273(266–282)	3418(3246–3501)	4234
Hubei	292	46	643(602–693)	96(55–146)	739(698–789)	3199(3027–3282)	6869
Liaoning	250	53	684(673–698)	33(22–47)	717(706–731)	3194(3022–3277)	9959
Heilongjiang	607	45	720(676–774)	110(66–164)	830(786–884)	3132(2960–3215)	6057
Inner Mongolia	340	32	528(518–539)	43(33–54)	571(561–582)	3028(2856–3111)	10882
Gansu	163	31	505(498–514)	20(13–29)	525(518–534)	2941(2769–3024)	3919
Ningxia	67	13	182(175–191)	15(8–24)	197(190–206)	2515(2343–2598)	6331
Jilin	351	27	399(389–410)	59(49–70)	458(448–469)	2433(2261–2516)	7619
Shandong	412	43	631(591–679)	96(56–144)	727(687–775)	2413(2241–2496)	9072
Qinghai	73	14	201(190–215)	37(26–51)	238(227–252)	2100(1928–2183)	5871
Henan	801	41	518(469–576)	136(87–194)	654(605–712)	2099(1927–2182)	5516
Hunan	187	36	469(459–480)	44(34–55)	513(503–524)	1933(1761–2016)	5913
Zhejiang	187	34	396(386–408)	47(37–59)	443(433–455)	1918(1746–2001)	11033
Beijing	61	8	103(91–118)	36(24–51)	139(127–154)	1859(1687–1942)	14889
Hebei	460	35	459(444–481)	78(63–100)	537(522–559)	1823(1651–1906)	6232
Shaanxi	332	22	309(286–337)	52(29–80)	361(338–389)	1809(1637–1892)	6884
Guangxi	151	25	380(367–394)	69(56–83)	449(436–463)	1768(1596–1851)	4920
Xinjiang	121	12	148(135–162)	60(47–74)	208(195–222)	1744(1572–1827)	7460
Fujian	92	22	318(311–327)	11(4–20)	329(322–338)	1743(1471–1726)	9310
Yunnan	167	25	355(347–369)	43(35–57)	398(390–412)	1735(1563–1818)	4062
Anhui	367	32	336(324–351)	66(54–81)	402(390–417)	1584(1412–1667)	5098
Guizhou	148	23	302(295–311)	21(14–30)	323(316–332)	1532(1360–1655)	3691
Chongqing	79	9	110(108–113)	8(6–11)	118(116–121)	1470(1298–1553)	6881
Jiangxi	186	20	248(236–262)	32(20–46)	280(268–294)	1436(1264–1522)	5120
Shanxi	291	19	234(221–248)	63(50–77)	297(284–311)	1203(1031–1286)	5606
Jiangsu	236	23	241(231–252)	52(42–63)	293(283–304)	1200(1022–1277)	12032
Sichuan	77	22	300(293–309)	13(6–22)	313(306–322)	1168(996–1251)	5230
Hainan	35	2	30(17–44)	53(40–67)	83(70–97)	1060(888–1143)	5675
Guangdong	137	13	163(153–174)	53(43–64)	216(206–227)	984(812–1067)	9430
Shanghai	125	1	7(5–20)	30(18–43)	37(25–50)	453(281–536)	14442
**National level**	**7030**	**758**	**10413 (9955–10977)**	**1563 (1105–2127)**	**11976 (11518–12540)**	**2010(1838–2093)**	**6750**

*According to the order from high to low of DALYs per 100,000.

During September 1, 2012 and August 31, 2013, the total number of YLLs caused by stroke in all age groups in 31 provinces was 10,413 (95% UI: 9955–10977), the total number of YLDs was 1,563 (95% UI: 1105–2127), and the total number of DALYs was 11,976 (95% UI:11518–12540) (Table 1).

**Fig 1:**
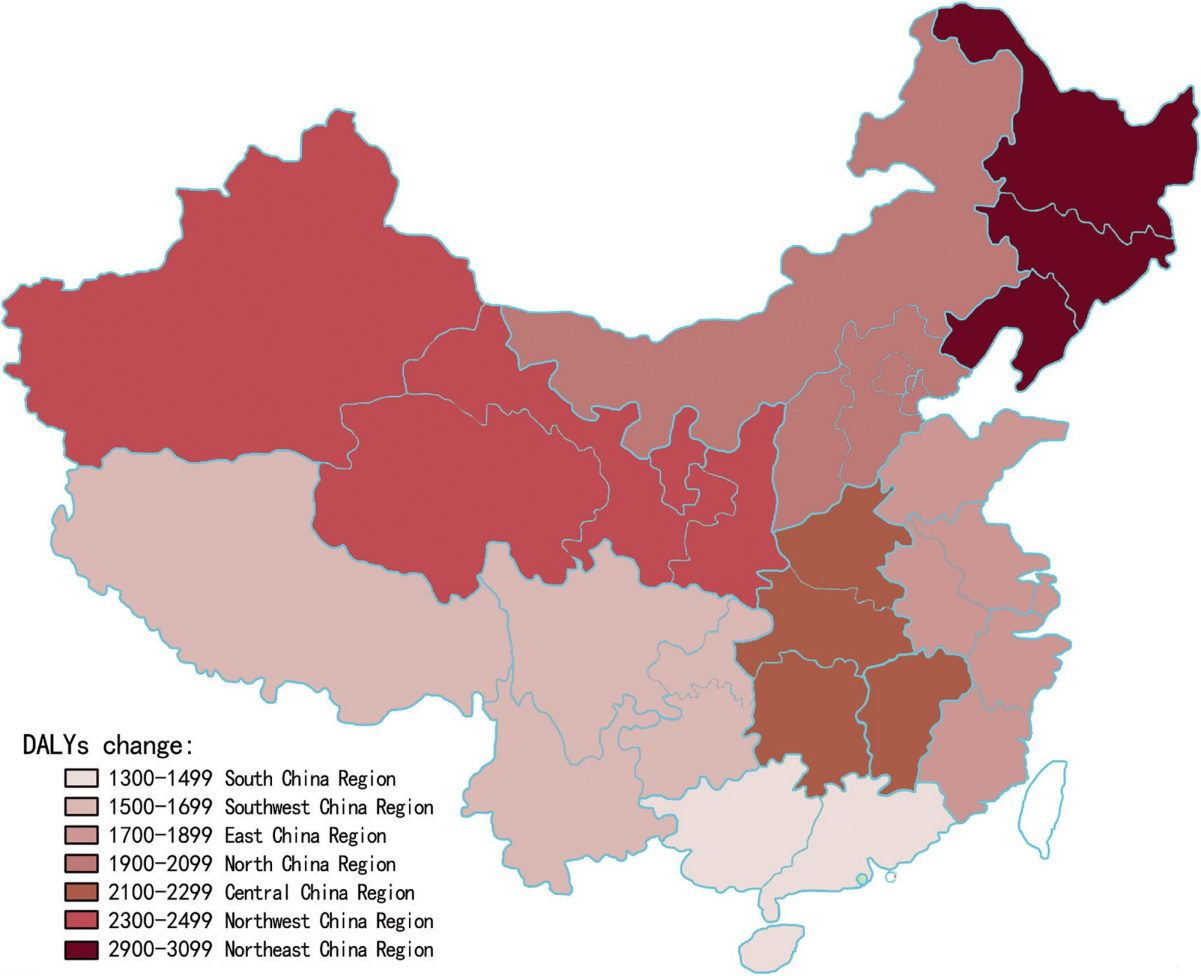
DALYs per 100,000 people in seven regions of China. The region with the highest DALYs rates was Northeast while the lowest DALYs rates were in South China.

From September 1, 2012 to August 31, 2013, YLLs rate per 100,000 people of all age groups per year for stroke nationwide was 1,748 (95% UI: 1576–1831), the YLDs rate was 262 (95% UI: 90–345) and the DALYs was 2,010 (95% UI: 1838–2093). Comparing the seven Chinese regions[19, 20], the region with the highest YLLs rates was Northeast, and the lowest rates were observed in South China; the region with the highest YLDs rates was North China while the lowest YLDs rates were observed in Southwest China; the region with the highest DALYs rates was Northeast while the lowest DALYs rates were in South China (Table 2 and Fig 1). 87% of DALYs were due to YLLs and the remaining 13% were due to YLDs. Generally, YLLs took up a dominant proportion of stroke-caused DALYs in China.

**Table 2:** YLLs, YLDs and DALYs per 100,000 people (95%UI) in seven regions nationwide, and the respective percentage*.

Regions (provinces)	YLLs (%)	YLDs (%)	DALYs
**Northeast China** (Heilongjiang, Jilin, Liaoning)	262 (2490–2745) (90%)	298 (126–381) (10%)	2960 (2788–3043)
**Northwest China** (Ningxia, Xinjiang, Qinghai, Shaanxi, Gansu)	1951 (1779–2034) (84%)	366 (194–449) (16%)	2317 (2145–2400)
**Central China** (Hubei, Hunan, Henan, Jiangxi)	1882 (86%)	308 (14%)	2190 (2018–2273)
**North China** (Beijing, Tianjin, Hebei, Shanxi, Inner Mongolia)	1761 (1589–1844) (84%)	331 (159–414) (16%)	2092 (1920–2175)
**East China** (Shandong, Jiangsu, Anhui, Zhejiang, Fujian, Shanghai)	1489 (1317–1572) (86%)	233 (61–316) (14%)	1722 (1550–1805)
**Southwest China** (Sichuan, Yunnan, Guizhou, Xizang, Chongqing)	1537 (1365–1620) (93%)	113 (59–196) (7%)	1650 (1478–1733)
**South China** (Guangdong, Guangxi, Hainan)	1044 (872–1127) (77%)	318 (146–401) (23%)	1362 (1190–1445)
**National level**	1748 (1576–1831) (87%)	262 (90–345) (13%)	2010 (1838–2093)

*According to the order from high to low of DALYs per 100,000.

During the study period, the rate of YLLs in rural areas was 1964(95%UI:1792–2047)/100,000, the rate of YLDs was 294(95%UI:122–377)/100,000, and the rate of DALYs was 2,258(95%UI:2086–2341)/100,000; the rate of YLLs urban areas was 1,533(95% UI: 1361–1616)/100,000, the YLDs was 229(95% UI: 57–312)/100,000, and the rate DALYs was 1,762(95% UI: 1590–1845)/100,000.

NESS-China has completed preliminary screening of 596,536 cases among permanent residents of all age groups at 155 survey sites in 31 provinces, among whom 3915 males and 3115 females were diagnosed with disability due to stroke before August 31, 2013; and 417 males and 341 females died due to stroke during September 1, 2012 and August 31, 2013.

**Fig 2:**
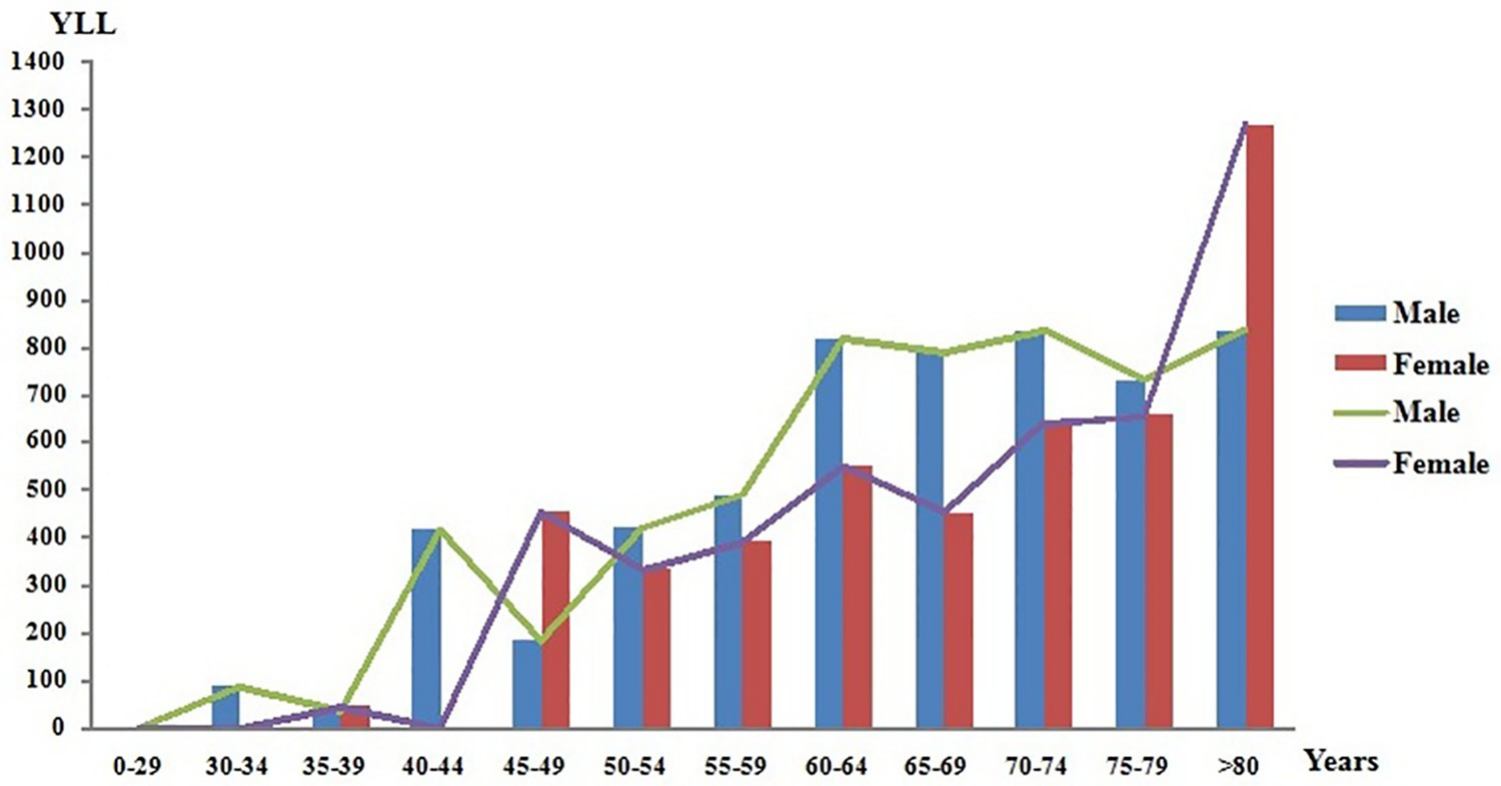
The gender and age stratification on the absolute value of YLLs. The columns indicate the number of YLLs; the lines show the tendency of YLLs at different age groups.

During September 1, 2012 and August 31, 2013, the absolute number of YLLs caused by stroke among all age groups in 31 provinces was 5638 males and 4776 females, the absolute number of YLDs was 868 (95% UI: 583–1210) among males and 694 (95% UI: 405–1033) among females, the absolute number of DALYs was 6506 (95% UI: 6221–6848) among males and 5470 (95% UI: 5181–5809) among females. The DALYs rate per 100,000 people was 2171(95% UI: 1999–2254) in males and 1848(95% UI: 1676–1931) in females. Among the 18 age groups analysed, the highest number of YLLs was observed in people aged over 80 years, especially in females (1267 compared to 835 in males) (Fig 2).

## Discussion

The present study is based on the data from NESS-China, the first epidemiological survey containing the large-scale samples and multiple centers established in 155 survey sites across 31 provinces specially aimed at studying cerebrovascular disease epidemiology[1, 2, 10, 18]. The survey was carried out primarily by trained investigators and professional neurologists who conducted face-to-face interviews and clinical/neurological examination of the study participants. Such cross-sectional surveys with large-scale samples and multiple centers by strict screening can ensure the reliability of stroke related epidemiological data, and can ensure that all quantitative stroke burden indices estimated on this basis are objective and accurate. This study adopts quality control specification at all survey sites nationwide, in order to ensure all quantitative indices are estimated on the same basis.

Our finding suggests that stroke is the main cause of YLLs in China, but not YLDs. This is consistent with the GBD 2010 Study results[12] that showed that YLDs caused by stroke mean ranked 25^th^ (27^th^ in China) among 289 disabling diseases and injures causing 1160 sequelae in all 21 GBD epidemiological regions[15, 21]. However, DALYs caused by stroke ranked 3^th^ in global among the 21 regions and 1^st^ in China (the top three diseases were stroke, ischemic heart disease and lung cancer)[1, 15]. Considering the rising number of people suffering from a stroke and the high related disability rate worldwide, YLDs continues to appear as an important metric for evaluating stroke related nonfatal health impact. It also appears to possess far-reaching significance for reducing disability rate and redeeming years of life lived with disability[22, 23].

The DALYs rates in China appeared to be higher compared to global GBD 2010 DALYs rate, with a significant difference (2,010 VS 1,484 [15, 16], p <0.001, adjusted to the same standard world population composition). Among the 21 GBD epidemiological regions, DALYs caused by stroke played the dominant role in the demographically and epidemiologically advanced regions such as East Asia (includes China) (Stroke Mean Rank 1 in 291 GBD diseases and injuries), High-income Asia Pacific, Southeast Asia, Eastern Europe, Central Europe, Central Asia and Western Europe. Meanwhile, the burden of diseases in the backward regions (science, economy, medical technology, demographic and epidemiological studies are lagging behind) such as sub-Saharan Africa, South Asia and Latin America, the leading volume of DALYs were due to communicable diseases and nutritional deficiencies, however not the cardiovascular and circulatory diseases includes stroke (Table 3). The heterogeneity across 21 regions in the burden of diseases highlighted how important it will be to make estimates at the national levels.

**Table 3:** DALYs per 100000 caused by stroke and stroke Mean Rank (95%UI) among the 21 GBD epidemiological regions [15] and China*.

Epidemiological Regions or Countries	DALYs	Stroke Mean Rank**
Eastern Europe	3914(3742–3997)	2(1–3)
Central Europe	3444(3272–3527)	2(1–3)
Central Asia	2661(2489–2744)	3(2–5)
East Asia	2192(2020–2275)	1(1–2)
Western Europe	2045(1873–2128)	3(2–5)
High-income North America	2035(1863–2118)	7(3–11)
North Africa and Middle East	2026(1854–2109)	4(3–8)
China***	2010(1838–2093)	1(1–3)
High-income Asia Pacific	1878(1706–1961)	1(1–2)
Southern Latin America	1875(1703–1958)	3(2–6)
Australasia	1722(1550–1805)	5(4–9)
Tropical Latin America	1720(1548–1803)	4(3–5)
Southeast Asia	1718(1546–1801)	1(1–2)
Central Latin America	1252(1080–1335)	11(7–14)
Andean Latin America	1098(926–1181)	11(7–17)
South Asia	1096(924–1179)	12(8–15)
Caribbean	939(767–1022)	3(1–4)
Oceania	783(611–866)	11(7–14)
Southern sub-Saharan Africa	626(454–709)	7(6–13)
Central sub-Saharan Africa	485(313–568)	14(12–16)
Eastern sub-Saharan Africa	470(298–553)	16(10–20)
Western sub-Saharan Africa	313(141–396)	16(11–22)
Global	1484(1312–1567)	3(2–5)

*According to the order from high to low of DALYs per 100,000.

**Ranking in 291 GBD diseases and injuries.

*** The data of our survey from “National Epidemiological Survey of Stroke in China” (NESS-China).

During September 1, 2012 and August 31, 2013, Through the comparison of seven different regions, rural and urban areas in this study[19, 20], the highest value of DALYs caused by stroke was found in the Northeast China and the rural areas while the lowest in South China and the urban areas. With reference to the gender and age stratification with burden of stroke, the value of DALYs in males was higher than in females generally. However, among the 18 age groups analysed, the highest number of YLLs was observed in people aged over 80 years, especially in females. As shown in the Fig 2, YLLs caused by stroke in females was significantly higher than in males for 45–49 years and over 80 years age group, maybe it was due to the perimenopausal period that caused females loss of estrogen to prevent cardio-cerebrovascular diseases. All of the above difference was statistically significant (p<0.001). The geographical, gender and age differences observed in stroke burden in China may be related to inter-regional demographic characteristic differences, regional medical treatment levels, population economy income conditions, etiological stroke subtypes and other risk factors, which means further analysis and assessments are needed[24].

Although morbidity and mortality rates have been declining in most high-income countries for the past two decades, they have been continuously rising in low and middle-income countries including China. This may be due to the life-style transformation, differences in medical conditions, clinical diagnosis and treatment levels, or the deficiency in high risk groups screening and management. The prevalence rate and burden of stroke is growing rapidly worldwide[25]. Currently, stroke related risk factors such as high blood pressure, diabetes mellitus, and hyperlipemia have also been rising[1, 26], and given the smoking rates have not significantly changed in developing countries, the morbidity rate, prevalence rate and death rate are all expected to rise in the near future[27].

China is facing rapid growth of this disease and the burden of stroke requires further investigation and study that would preferably include multiple centers, and large-scale assessments of various factors. Establishing a national stroke register is an urgent issue. Dynamic epidemiology monitoring, clinical diagnosis and treatment of cerebrovascular diseases, screening and management of disease and high-risk population, study and promotion of suitable intervention technologies, are all indispensable for constructing a useful and effective system suitable for coping with cerebrovascular diseases in view of existing national conditions. This would furthermore help strengthen the implementation of primary and secondary prevention strategies, so as to reduce the huge health impact of stroke in China on individual and societal level[28]. This study may serve as a useful guide for control planning strategy, prevention and treatment integration, and dynamic monitoring reinforcement in differently developed regions[1, 29, 30]. The updates would not only provide a mechanism both to assess the latest cross-sectional surveys’ evidences but also to promote accountability of health systems for achieving reductions in the burden of stroke. Furthermore, despite this complexity and diversity, important medical health and public services fundamental challenges are readily identifiable for which technologies and knowledge exist to substantially reduce or eliminate their impact on burden of disease rankings. The sustained commitment of governments, medical workers and the public health community to do so is crucial, on the basis of the essential health intelligence that regular burden of stroke updates can provide promptly.
